# Targeted CRISPR activation and knockout screenings identify novel doxorubicin transporters

**DOI:** 10.1007/s13402-023-00847-0

**Published:** 2023-07-31

**Authors:** Yufeng Li, Minkang Tan, Shengnan Sun, Elena Stea, Baoxu Pang

**Affiliations:** Department of Cell and Chemical Biology, Leiden University Medical Center, Leiden, Netherlands

**Keywords:** CRISPR Screening, Anti-cancer, Doxorubicin, Transporters

## Abstract

**Purpose:**

Tissue-specific drug uptake has not been well studied, compared to the deeper understanding of drug resistance mediated by the cellular efflux system such as MDR1 proteins. It has been suggested that many drugs need active or defined transporters to pass the cell membrane. In contrast to efflux components induced after anti-cancer drugs reach the intracellular compartment, drug importers are required for initial drug responses. Furthermore, tissue-specific uptake of anti-cancer drugs may directly impact the side effects of many drugs when they accumulate in healthy tissues. Therefore, linking anti-cancer drugs to their respective drug import transporters would directly help to predict drug responses, whilst minimizing side effects.

**Methods:**

To identify drug transporters of the commonly used anti-cancer drug doxorubicin, we performed focused CRISPR activation and knockout genetic screens targeting all potential membrane-associated transporters and proteins. We monitored the direct uptake of doxorubicin by fluorescence-activated cell sorting (FACS) as the screening readout for identifying transporters/proteins directly involved in doxorubicin uptake.

**Results:**

Integrating the data from these comprehensive CRISPR screenings, we confirmed previously indicated doxorubicin exporters such as ABCB1 and ABCG2 genes, and identified novel doxorubicin importer gene SLC2A3 (GLUT3). Upregulation of SLC2A3 led to higher doxorubicin uptake and better cell killing, indicating SLC2A3 could be a new marker to predict doxorubicin drug response and minimize side effects for the personalized application of this conventional chemotherapeutic drug.

**Conclusions:**

Our study provides a comprehensive way for identifying drug transporters, as exemplified by the commonly used anti-cancer drug doxorubicin. The newly identified importers may have direct clinical implications for the personalized application of doxorubicin in treating distinct tumors. Our results also highlight the necessity of combining both CRISPR knockout and CRISPR activation genetic screens to identify drug transporters.

**Supplementary Information:**

The online version contains supplementary material available at 10.1007/s13402-023-00847-0.

## Introduction

Precisely treating the diseased cells or tissues whilst exhibiting minimal side effects on healthy bystander cells has always been the ultimate goal of medicine. This is especially challenging for chemotherapeutic drugs that treat cancer, as these drugs’ effectiveness often coincides with notorious side effects. There can be several ways to reach this therapeutic window. First, by selecting drugs that could reach the target cells better, the total dose could be reduced to minimize side effects. Second, choosing drugs that do not affect normal tissues but only affect the target cells, exemplified by modern targeted therapy drugs like imatinib (Gleevec), which only targets a specific protein kinase in tumor cells [1], should decrease toxicity.

Although many drugs are very effective for different tumors, de novo resistance does exist. Much research has been done since the realization of drug resistance, and there are many mechanisms proposed to explain the drug resistance, such as up-regulation of drug transporters to pump out drugs, bypassing the original drug target by the tumor cells, or boosting the cell survival pathways [2–5]. However, to exert their effects, most drugs (except for antibody-based drugs) first need to reach the inside of the cell. It is generally assumed that most drugs pass the cell membrane by passive diffusion [6, 7]. However, recent research indicates that carrier-mediated uptake of drugs may be more common and important than we thought [6, 7]. But only limited systematic research has been done to define the uptake mechanisms of chemotherapeutic drugs, where SLC-specific CRISPR–Cas9 knockout genetic screening has been used to identify potential drug transporters [8]. However, many potential drug transporters are lowly expressed or not even expressed at all in a given cell type, where deleting these genes by CRISPR–Cas9 knockout genetic screenings will not be able to show a phenotype, therefore making it hard to evaluate potential drug transporters comprehensively.

To evaluate all potential drug transporters in a comprehensive way, we created focused CRISPR–Cas9 knockout (CRISPRko, to induce loss of function of endogenous genes) [9–12] and CRISPR/dCas9 activation (CRISPRa, to up-regulate endogenous genes) [13–16] libraries targeting all known transporters from the human genome. We integrated the results from the two screening systems to identify new transporters for different drugs. Anthracycline family drugs are amongst the most widely used anti-cancer drugs and include several analogs that exert different effectivity in solid and non-solid tumors [17, 18]. The tissue distribution of these drugs is very different [19, 20], which often coincides with side effects observed in the clinic, therefore it is possible that different drug importers might play important roles in the efficacy and side effects of these drugs.

We performed CRISPRko and CRISPRa genetic screens to identify the potential transporters for doxorubicin, the most widely used anthracycline drug with strong side effects such as cardiotoxicity [18, 21–23]. Integrating the data from these comprehensive CRISPR screenings, we confirmed drug exporters for doxorubicin such as ABCB1 and ABCG2 genes, and identified novel doxorubicin importer gene SLC2A3 based on the CRISPRa genetic screens. Upregulation of SLC2A3 led to higher drug uptake and better cell killing, indicating SLC2A3 could be a new marker to predict doxorubicin drug response and minimize side effects for the personalized application of this conventional chemotherapeutic drug. These results also highlight the necessity of combining both CRISPRko and CRISPRa genetic screens for the identification of drug transporters.

## Materials and methods

### Cell lines and cell culture

K562 cells were cultured in RPMI 1640 (Gibco) supplemented with 10% fetal bovine serum (Biowest), L-Glutamine (Gibco), and 1% Penicillin-Streptomycin (Gibco), at 5% CO2 in a humidified 37 °C incubator. 293T cells were cultured in DMEM (Gibco) supplemented with 10% fetal bovine serum (Biowest), L-Glutamine (Gibco), and 1% Penicillin-Streptomycin (Gibco). For stable dCas9-VP64-MS2-P65-HSF (K562/SAM) cell line generation, the wild-type K562 cell line was transduced with lentivirus generated using the Lenti MS2-P65-HSF1-Hygro plasmid at an MOI of 0.3 (Addgene #61426) and selected with 20 µg ml^− 1^ hygromycin (InvivoGen) for at least 1 week. Then dCas9-VP64 lentivirus was transduced into K562-MS2-P65-HSF1 cell line, and selected with blasticidine at 8 µg ml^− 1^. Single cells were seeded in 96-well plates and expanded.

### Reagents

Doxorubicin was obtained from Pharmachemie (the Netherlands). Aclarubicin was obtained from Santa Cruz (USA). Daunorubicin was obtained from Sanofi-Aventis (the Netherlands). Epirubicin was obtained from Accord Healthcare Limited (UK). Idarubicin was obtained from Pfizer (USA). All of these drugs were dissolved according to the manufacturer’s formulation.

### Design of the libraries

The RefSeq IDs of potential transporter genes were extracted from TransportDB 2.0 [24] and converted into gene symbols with DAVID [25].

For CRISPRko sgRNA library design, GUIDES web tool was used to target 952 potential transporter genes in the list, and on average 12 individual guides were prioritized by GUIDES for each gene [26]. Furthermore, 10 individual guides were designed to target 58 essential genes from K562 as positive controls and 400 human genome non-targeting sgRNAs from the GeCKO v2.0 library were added as negative controls [12, 27]. The designed CRISPR knockout library sequences (20 nt protospacer) were inserted into the cloning sequence backbone: GGAAAGGACGAAACACCG-[20 nt protospacer]-GTTTTAGAGCTAGAAATAGCAAGTTAAAATAAGGC and sent for oligo pool synthesis.

For CRISPRa sgRNA library design, the previous guide designs targeting 300 bp upstream regions from the transcription start sites of 977 potential transporter genes in the list were selected [15]. On average, 7 individual guides were included for each gene. 400 human genome non-targeting sgRNAs from the GeCKO v2.0 library were added as negative controls. The designed CRISPR activation library sequences (20 nt protospacer) were inserted into the cloning sequence backbone: GGAAAGGACGAAACACCg-[20 nt protospacer]- GTTTTAGAGCTAGGCCAACATGAGGATCACCCATG and sent for oligo pool synthesis.

### Construction of the CRISPRko and CRISPRa libraries

Construction of the libraries was performed as previously described [28]. Oligos with the CRISPRko and CRISPRa sgRNA (20 nt protospacer) sequences and flanking cloning sequences were ordered as oligo pools from Genscript.

For the CRISPRko library, the synthesized single-strand oligo pool was directly assembled with the digested vector products by Gibson Assembly. The lentiCRISPRv2-puro plasmid (Addgene, Plasmid #98290) was digested with Esp3I (Anza™ 13) and dephosphorylated using FastAP (Thermo Scientific™, EF0651) for generating the CRISPRko library. The resulting linearized vector products were further size-selected and gel-purified using QIAGEN MinElute column and used for library cloning using Gibson Assembly. The assembly mix was made using 200 ng of digested lentiCRISPRv2-puro vector, 6 ng CRISPRko oligo pool (at molar ratio 1:10) and 10 µl of 2× Gibson Assembly Master Mix for a final volume of 20 µl. The assembly mix was incubated at 50 °C for 60 min, and in total 10 reactions were pooled for making the full library with good coverage. The Gibson Assembly mix was purified by isopropanol precipitation and resuspended in 5 µl water, from which 1 µl of the products were electroporated into 25 µl of Endura electrocompetent cells (Endura, 60242). Five individual electroporation reactions were pooled and grown in 5 ml recovery medium for 1 h. Then 5 µl media from the 5 ml LB culture was used to perform a serial dilution to determine the electroporation efficiency and thus the library coverage, which was aimed to be at least 300-fold. The rest was further cultured in 1000 ml LB medium with 100 µg ml^–1^ carbenicillin overnight. The plasmid libraries were extracted using the QIAGEN Maxiprep Kit and verified by next-generation sequencing.

For the CRISPRa library, the synthesized oligo library was first amplified by PCR using the following primer:

CRISPRa- Forward primer: GTAACTTGAAAGTATTTCGATTTCTTGGCTTTATATATCTTGTGGAAAGGACGAAACACC;

CRISPRa- Reverse primer: ATTTTAACTTGCTAGGCCCTGCAGACATGGGTGATCCTCATGTTGGCCTAGCTCTAAAAC;

PCR procedures using NEBNext High-Fidelity 2× PCR Master Mix were: 98 °C for 30 s, 15 cycles of 98 °C for 10 s, 68 °C for 30 s and 72 °C for 30 s, and 72 °C for 5 min. For each reaction, 10 ng of the oligo pool was used for a 100 µl PCR reaction, and 20 reactions per library were pooled. The pooled PCR products were further size-selected and gel-purified using QIAGEN MinElute column. The lenti-sgRNA(MS2)-Puro plasmid (Addgene, Plasmid #73797) was digested with Esp3I (Anza™ 13) and dephosphorylated using FastAP (Thermo Scientific™, EF0651). The resulting linearized vector products were further size-selected and gel-purified using QIAGEN MinElute column and used for library cloning using Gibson Assembly. The assembly mix was made using 200 ng of digested lenti sgRNA(MS2)-Puro plasmid vector, 11.5 ng insert DNA (at molar ratio 1:10) and 10 µl of 2× Gibson Assembly Master Mix for a final volume of 20 µl. The downstream library construction and production steps were the same as for the CRISPRko library.

### Lentivirus production and MOI determination

For generating MS2-P65-HSF1 (MPH), dCas9-VP64 and lentiSAMv2-Puro virus, the 293T cells were grown in 6 well plates. When cells reached 50% confluency, they were transfected with the mix of 1 µg of Lenti MS2-P65-HSF1-Hygro plasmid or dCas9-VP64-blast plasmid or lentiSAMv2-Puro plasmid, 0.6 µg of psPAX2, 0.4 µg of pCMV-VSV-G, and 6 µg PEI (Polyscience, 23966) in 50 µl of serum-free medium. Media supernatant containing the virus particles was collected on the third day after transfection.

For packaging lentivirus of the screening libraries, the 293T cells were grown in 15 plates of 15 cm^2^ dishes for each library. For each dish, cells of 50% confluency were transfected with the mix of 30 µg of library plasmids, 20 µg of psPAX2, 10 µg of pCMV-VSV-G, and 180 µg PEI in 1 ml of serum-free medium. Media were refreshed the day following transfection. The medium supernatant containing virus particles was collected on the second and third days after transfection. Pooled medium supernatant was further concentrated using ultracentrifugation (4 °C, 10,000 g, 2 h). The virus titer was determined by making serial dilutions (10^–3^ to 10^–10^) of 10 µl of frozen virus supernatant to infect 293T cells. Two days after infection, cells were selected with 2 µg ml^–1^ puromycin for an additional 7 days. The virus titer was then calculated based on the survival colonies and the related dilution.

### Pooled CRISPRko and CRISPRa screen

The K562 cells were transduced with the lentiviral CRISPRko library, and K562/SAM stable cells were transduced with the lentiviral CRISPRa libraries respectively by spin-infection to achieve an initial library coverage of at least 500-fold at an MOI of ~ 0.3. For spin-infection, 3 × 10^6^ cells in each well of a 12-well plate were infected in 1 ml of medium containing 8 µg ml^–1^ of polybrene and the virus. In total, four 12-well plates were used for each screening to infect a total of 1.5 × 10^8^ cells. Then, two days after infection, successfully transduced cells were selected with 2 µg ml^–1^ puromycin (Biomol) for a further 3 days. Cells were harvested and dead cells were then removed with Histopaque-1077 (Sigma) by centrifuging cells at 400 g for 30 min at room temperature. After live cell enrichment, an aliquot of 10^8^ cells infected with either the CRISPRko or CRISPRa libraries were frozen as the control population. From the same cell population, another aliquot of 10^8^ K562 cells were treated with 2 µM doxorubicin for 2 h. Then cells were sorted based on their fluorescence intensity as a surrogate for direct doxorubicin uptake. In total 2 × 10^6^ cells that fell in the lowest 10% fluorescence intensity (L10) and highest 10% fluorescence intensity (H10) were FACS sorted respectively, and were frozen for downstream processing. For each biological replicate experiment, lentivirus was made freshly, and the infection and doxorubicin treatment were repeated.

### Genomic DNA extraction, library preparation, and sequencing

Genomic DNA was isolated from frozen cell pellets using the QIAmp DNA Blood and Tissue Maxi Kit (Qiagen). The sgRNA spanning region was amplified from purified genomic DNA, with primers containing the Illumina adapters and indices using the one-step PCR reaction. For each 100 µl PCR reaction, 10 µg of genomic DNA, 50 µl of 2× NEBNext High-Fidelity master mix, 2 µl of 10 µM forward and reverse primers were used respectively [15]. In total 5 PCR reactions with different pairs of primer were used and pooled, assaying in total of 50 µg of genomic DNA. PCR procedures were: 98 °C for 30 s, 19 cycles of 98 °C for 10 s, 68 °C for 30 s and 72 °C for 30 s, and 72 °C for 5 min. The PCR products were further size-selected and gel-purified using the QIAGEN MinElute column and confirmed using a High Sensitivity Bioanalyzer DNA Kit (Agilent). Then the libraries were sequenced on the Illumina HiSeq4000 platform.

### Pooled CRISPRko and CRISPRa screen analysis

Cutadapt 3.4 was used to extract the unique 20 nt protospacer sequences from Illumina paired-end reads by trimming out the U6 promoter overlapping sequence from the 5’ end and the scaffold sequence from the 3’ end. For the CRISPRko sgRNA library, the following sequences were used: ATCTTGTGGAAAGGACGAAACACCG (U6 promoter), GTTTTAGAGCTAGAAATAGC (scaffold). For the CRISPRa sgRNA library, the following sequences were used: ATCTTGTGGAAAGGACGAAACACCG (U6 promoter), GTTTTAGAGCTAGGCCAACA (scaffold). Trimmed reads were then aligned to the custom screen libraries using BWA [29]. To quantify each sgRNA abundance, from the same pair-end read, read with MAPQ score over 30 on one end and MAPQ score at least over 10 on the other end was accounted as valid. MAGeCK RRA was used to identify the sgRNAs of enriched/depleted significantly from comparisons between the FACS sorted highest 10% fluorescence intensity (H10) cells or lowest 10% fluorescence intensity (L10) cells and unsorted control cell population [30]. The sgRNA enrichment/depletion performance was further aggregated to identify positively/negatively selected genes in the comparison robustly.

### Generation of clones

Single-guide RNAs targeting the promoter regions of ABCB1 and SLC2A3 genes were selected from the pooled CRISPRa screen analysis. The guide RNA sequence was cloned into lentiSAMv2-Puro plasmid containing the gRNA scaffold and dCas9 sequence, and lentivirus was made as previously described. Then K562/SAM stable cells were transduced with the virus containing the respective guide RNAs and then selected using puromycin (2 µg ml^–1^). Single clones of cells were picked and verified using PCR and Sanger sequencing. The sequences of the gRNAs are listed in Supplementary Table 1.

### FACS analysis of drug uptake

The K562 ABCB1-CRISPRa, K562 SLC2A3-CRISPRa clones and 293T SLC2A3-CRISPRa bulk cells were treated with the respective drugs at 2 μM for 2 h, and then analyzed by FACS as the indication of drug uptake.

### Cell viability assay

The K562 ABCB1-CRISPRa and K562 SLC2A3-CRISPRa clones were seeded in 96-well plates at the density of 5 × 10^4^ cells per well. Then, doxorubicin or other anthracycline drugs were added to the cells at a series of concentrations indicated. After 72 h of treatment, CellTiter-Blue (Promega) was used to quantify the cell viability according to the manufacturer’s protocol.

### Western blot

Cells were washed with cold PBS and lysed in 1× RIPA buffer (Thermo Scientific) containing the protease inhibitor cocktail (Roche). Then the cell lysate was sonicated and the protein concentration in the supernatant was determined using the Pierce BCA protein assay kit (Thermo Scientific). Equal amounts of proteins were separated via 8% SDS-PAGE. Proteins were transferred to PVDF membranes, blocked with 5% nonfat milk in PBST for 1 h at room temperature, probed with primary antibodies against ABCB1 (CST 13342, 1:1000), SLC2A3 (Abcam ab191071, 1:1000), and Vinculin (Merck V9131, 1:5000; as the loading control) overnight at 4 °C. Membranes were washed with PBST three times, incubated with the appropriate secondary antibodies (1:10000 dilution) for 1 h at room temperature, and then washed with PBST three times. The resulting signal was visualized by Bio-Rad ChemiDoc Imaging System using the Pierce enhanced chemiluminescence (ECL) Plus Western Blotting Substrate (Thermo Scientific).

### Quantitative PCR

Total RNA was isolated from 10^6^ cells using ISOLATE II RNA Mini Kit (Bioline, BIO-52073) according to the manufacturer’s instructions. The cDNAs were synthesized from 500 ng of total RNA using a SuperScript™ IV VILO™ Master Mix (Invitrogen, 11756050), and were analyzed using SensiFAST SYBR No-ROX Kit (Bioline, BIO-98020) with primers for ABCB1, SLC2A3, and GAPDH (a housekeeping gene) on Biorad CFX Opus 384 Real-Time PCR Systems system. The sequences of the primers are listed in the Supplementary Table 1.

### Statistical analyses

For determining the significance of differences in the experimental data, the Student’s t-test (two-tailed), one-way analysis of variance, and the Mann‒Whitney U test were performed using GraphPad Prism version 8 software. P values less than 0.05 were considered to be significant.

## Results

### Construction of custom CRISPRko and CRISPRa libraries

**Fig. 1:**
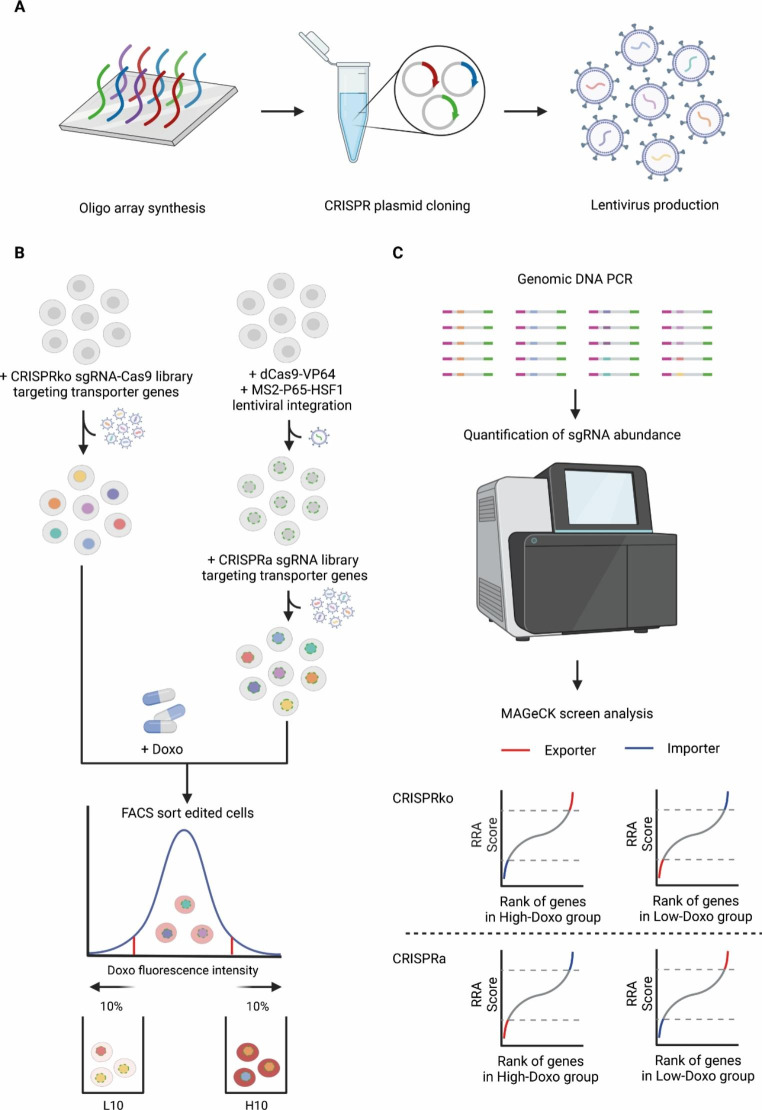
Workflow of the transporter-targeting CRISPRko and CRISPRa screenings. **A**. Two custom oligo pools were designed and synthesized for CRISPRko and CRISPRa library plasmid construction. Synthesized oligo pools were cloned into the digested CRISPRko or CRISPRa plasmid backbones. Constructed CRISPRko or CRISPRa plasmid library was transfected into 293T cells together with packaging plasmids to produce lentiviral CRISPRko and CRISPRa library. **B**. Outline of the drug uptake screenings. The K562 cells with CRISPRko or CRISPRa library were treated with 2 µM doxorubicin for 2 h before FACS sorting. The cells falling in the lowest 10% fluorescence intensity (L10) and highest 10% fluorescence intensity (H10) were sorted for downstream processing. **C.** The sgRNA spanning region was PCR amplified from total genomic DNA that was isolated from sorted or control cells for NGS sequencing. A custom computational workflow was used to quantify the sgRNA abundance. MAGeCK was used to identify positively or negatively selected genes for doxorubicin uptake or export with the cutoff of RRA score < 0.001

An unbiased way to search for drug transporters in a systematic and comprehensive way is to use a genome-wide screening system, such as insertional mutagenesis, CRISPRko or CRISPRa genetic screenings [9, 10, 31, 32]. However, such screenings are intrinsically noisy [33]. If other confounding phenotypes are dominant in the screen readout, the drug transporters may not be identified during such screenings, since the drug might also be able to diffuse into the cells [10, 34]. As an alternative, we constructed a custom and dedicated CRISPRko library (for gene silencing, which would help to identify expressed transporters that determine the drug uptake in different cell types), as well as CRISPRa library (for gene activation, which would identify both expressed and not expressed potential transporters that may be involved in the drug uptake), specifically targeting the known transporters and transporter associated proteins (TransportDB 2.0) [24]. In this way, focused screening systems were made to study all potential transporters, irrespective of their expression level in certain cell lines (Supplementary Fig. 1). In total 1313 potential importers from TransportDB 2.0 were targeted for design, and the respective CRISPR knockout (952 genes were targeted) and activation libraries (977 genes were targeted) were generated. For the transporter CRISPRko library, 12 guides per gene were designed to inactivate the targeting transporters based on published algorithm [26]. For the transporter CRISPRa library, the 300 bp upstream regions of the transcription start sites of the 977 potential importers were used to design the CRISPR guide RNAs [15]. The two libraries were then assembled according to the protocol [33] (Fig. 1A).

Doxorubicin, like many other anthracycline drugs, is autofluorescent [17], which allows the monitoring of direct drug transport by fluorescence-activated cell sorting (FACS). K562 cells either infected with the transporter CRISPRko or CRISPRa libraries were treated with 2 μM doxorubicin for 2 h. Then cells were sorted based on their fluorescence intensity as a surrogate for direct doxorubicin uptake. Cells that fell in the lowest 10% fluorescence intensity (L10) and highest 10% fluorescence intensity (H10) were FACS sorted, respectively, and subjected to downstream processing (Fig. 1B). Genomic DNA from the respective populations was isolated, and CRISPR guide RNA sequences were PCR amplified and subjected to next-generation sequencing. The diversity and abundance of the guide RNAs in different FACS-sorted populations were compared to the initial population before the FACS sort, and enriched and depleted guide RNAs were calculated using MAGeCK (Fig. 1C) [30].

**Fig. 2:**
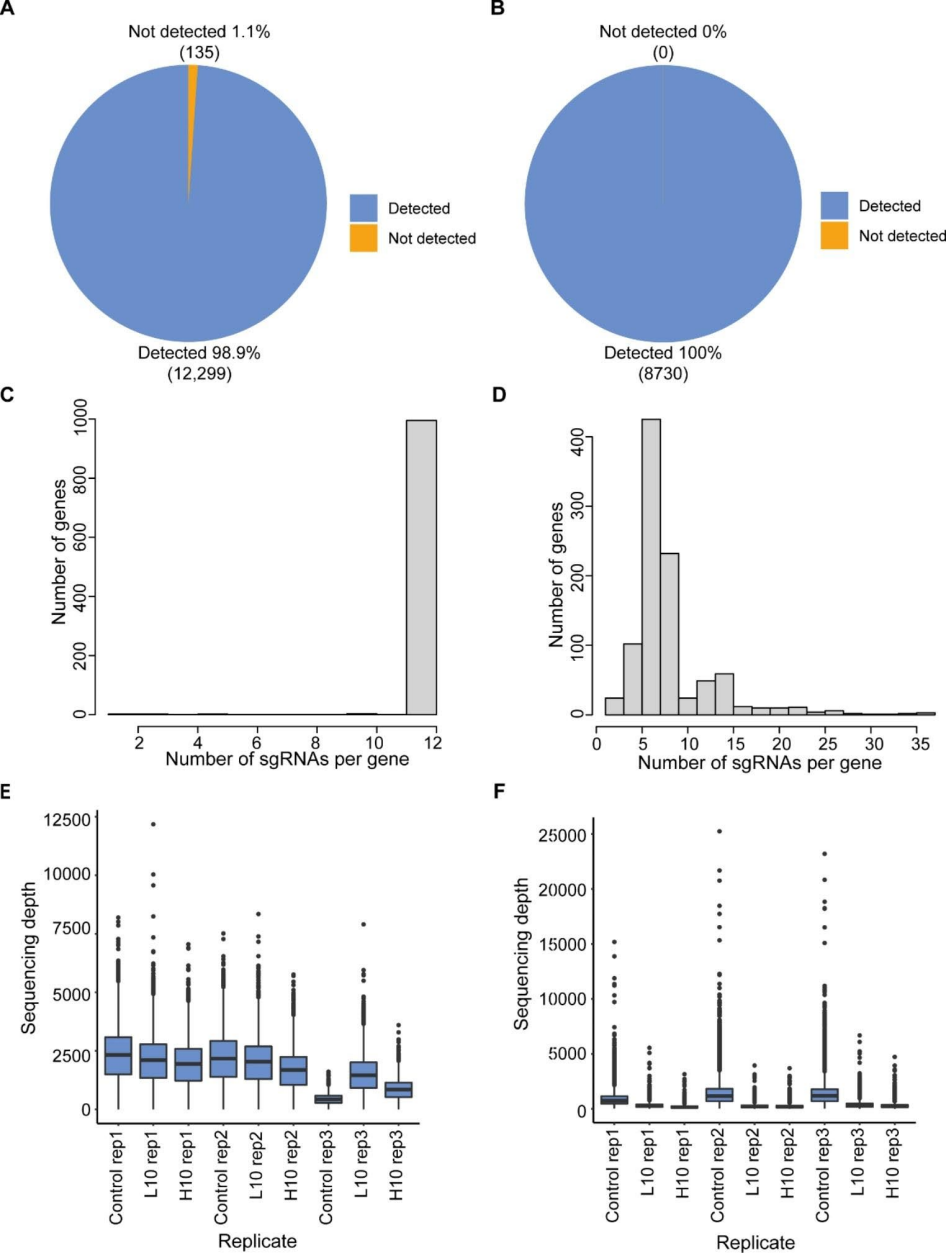
Quality control of transporter-targeting CRISPRko and CRISPRa libraries. **A-B**. Piechart of the percentage of detected or undetected sgRNA in cells infected with CRISPRko (A) or CRISPRa libraries (B). The detected or undetected sgRNAs were indicated in different colors. **C-D**. Histogram shows the distribution of sgRNAs per gene from CRISPRko (C) and CRISPRa (D) libraries by sequencing. The y axis represents the number of genes. The x axis indicates the specific number of sgRNAs. **E-F**. Boxplot indicates the distribution of read coverage per sgRNA in all CRISPRko (E) and CRISPRa (F) screening samples. The x axis represents the different screening replicates. The y axis represents the read depth of each sgRNA

Designed CRISPRko and CRISPRa libraries were first verified by sequencing. We recovered 98.9% of the designed guide RNAs from CRISPRko library (Fig. 2A), and 100% of the designed guide RNAs from CRISPRa library (Fig. 2B). In the CRISPRko library, on average 12 guides per gene were recovered (Fig. 2C), and in the CRISPRa library at least 5 guides were recovered to target each gene promoter for the majority of the target genes (Fig. 2D). For each sequencing sample, an average 500× and 200× sequence depth per guide were reached for CRISPRko (Fig. 2E) and CRISPRa (Fig. 2F) screenings respectively. The correlations of all the samples from both CRISPRko and CRISPRa screenings were good (Supplementary Fig. 2).

### Screening direct doxorubicin transport using CRISPRko and CRISPRa libraries

**Fig. 3:**
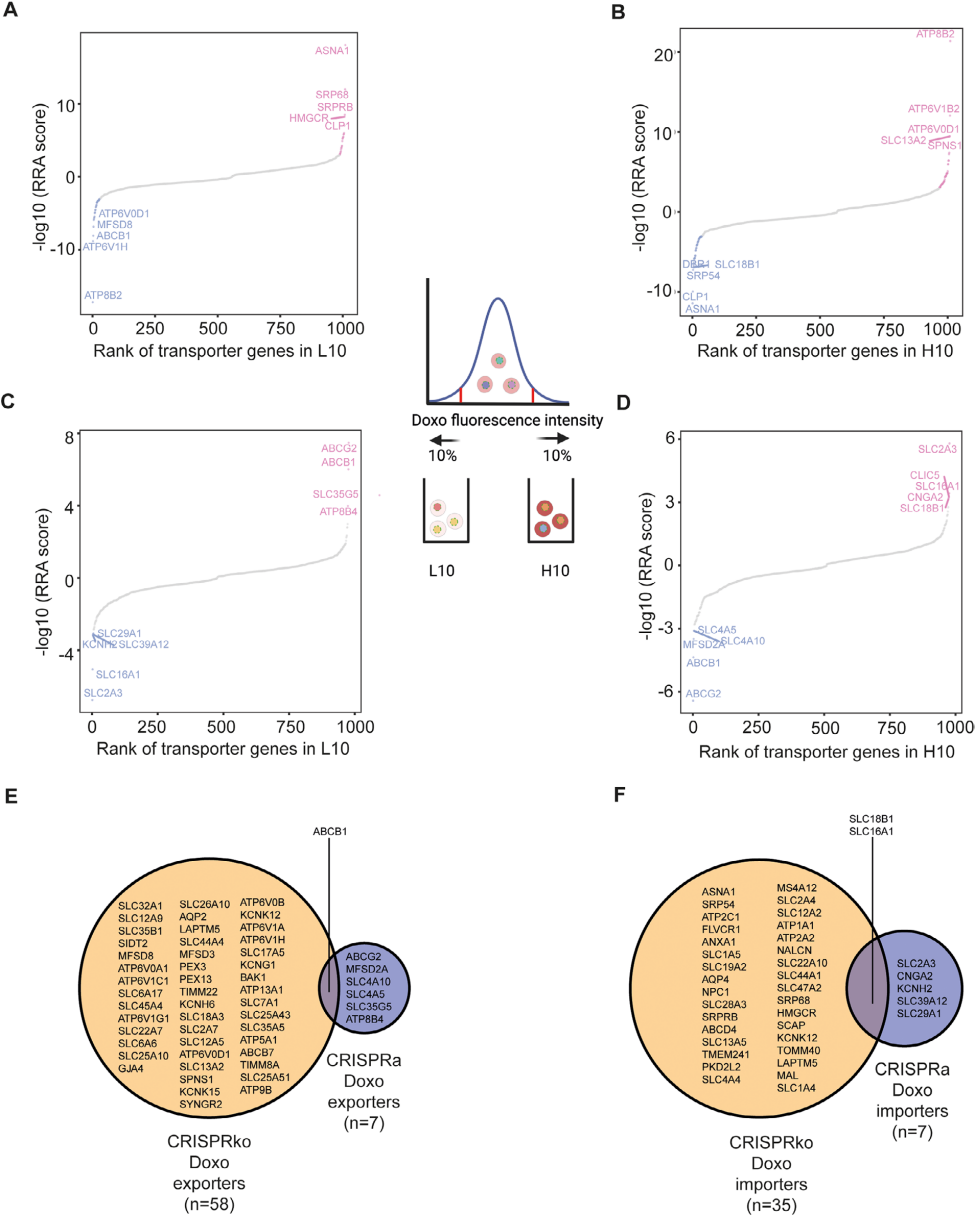
Identification of potential genes involved in doxorubicin transport from CRISPRko and CRISPRa screenings. **A**. Potential hits from L10 population in CRISPRko screening. **B**. Potential hits from H10 population in CRISPRko screening. **C**. Potential hits from L10 population in CRISPRa screening. **D**. Potential hits from H10 population in CRISPRa screening. The y axis represents MAGeCK RRA score. The x axis represents the ranking of the genes. The positively selected genes were indicated in red and negatively selected genes were indicated in blue using the cutoff of MAGeCK RRA score < 0.001. The top 5 genes in both selection directions were highlighted by their gene symbols. **E**. Venn diagram shows the overlapping hits potentially involved in doxorubicin drug export from CRISPRko and CRISPRa screens. **F**. Venn diagram shows the overlapping hits potentially involved in doxorubicin drug import from CRISPRko and CRISPRa screens

In the CRISPRko screening, the enriched genes in the L10 population represent the potential membrane proteins involved in doxorubicin importing (Fig. 3A, top-right corner in red and Supplementary Table 2). More than 10 potential membrane proteins involved in doxorubicin import were identified. The top hit is gene ASNA1, an ATPase and a component of transmembrane domain (TMD) recognition complex (TRC) that is involved in the post-translational delivery of tail-anchored (TA) proteins from the cytosol to the endoplasmic reticulum (ER) [35]. On the contrary, the depleted genes in the L10 population represent the 10 potential membrane proteins involved in doxorubicin exporting (Fig. 3A, lower-left corner in blue and Supplementary Table 3). Multiple ATPases were among the top hits. In addition, the well-known multi-drug resistance exporter gene ABCB1 which is frequently identified in different genetic screenings for drug resistance [2, 34], was also identified. The ABCB1 gene was not ranked the highest, possibly because the screening was done under a short period of doxorubicin exposure and the ABCB1 gene needs to be upregulated in response to drug exposure.

When the H10 population from the CRISPRko screening was analyzed, similar groups of genes were identified. Here the enriched genes represent 44 potential membrane proteins involved in drug exporting (Fig. 3B, top-right corner in red and Supplementary Table 3). It is interesting to see that gene ATP8B2 ranked amongst the top hits in this population, whilst also ranked within the top hits of the potential membrane proteins involved in drug exporting in L10 population (Fig. 3A, lower-left corner in blue). The depleted genes in the H10 population represent 42 potential membrane proteins involved in drug importing (Fig. 3B, lower-left corner in blue and Supplementary Table 2), which also includes gene ASNA1 among the top hits. This gene was also enriched as the top hit involved in drug importing in the L10 group (Fig. 3A, top-right corner in red). The fact that many same gene hits were indicated for the same potential function from different populations suggests that the CRISPRko screening was robust and would reliably identify potential proteins that are involved in doxorubicin transport.

Lowly expressed genes are not feasible to be studied using CRISPR knockout. On the contrary, CRISPR activation would allow the identification of lowly expressed genes which would play a role in drug transport. Therefore we expect that additional novel genes would be identified compared to the CRISPRko screening. From the CRISPRa screenings with a similar experimental set-up, the enriched genes in the L10 population represent the 4 potential membrane proteins involved in drug exporting (Fig. 3C, top-right corner in red and Supplementary Table 3). Again the well-known drug transporter genes ABCG2 and ABCB1 were significantly enriched [2, 34, 36], among some other genes such as genes SCL35G5 and ATP8B4, indicating upregulation of these genes led to less drug accumulation. On the other side, the SLC2A3 gene, among some other solute carrier transporter genes, were depleted in the L10 population, suggesting these may be the drug importers (Fig. 3C, lower-left corner in blue and Supplementary Table 2).

From the CRISPRa screenings, the enriched genes in the H10 population would indicate 4 potential genes involved in drug importing. The top hit, among some other solute carriers, was the SLC2A3 gene (Fig. 3D, top-right corner in red and Supplementary Table 2), confirming the results from similar analyses in a different population (Fig. 3C, lower-left corner in blue). The top depleted hits from H10 population, which indicates drug exporting roles, were the ABCG2 and ABCB1 genes (Fig. 3D, lower-left corner in blue and Supplementary Table 3), which were also seen in similar analyses in the L10 population (Fig. 3C, top-right corner in red). All these mutually confirming data indicate that the CRISPRa screening identified reliable hits involved in drug exporting and importing.

We further grouped all the potential genes involved in drug exporting identified from CRISPRko and CRISPRa screenings respectively, and compared them together. Only the ABCB1 gene was jointly identified from the two different types of screenings (Fig. 3E). A similar analysis was also performed integrating the potential genes involved in drug importing from both screenings. Only two solute carrier genes SLC16A1 and SLC18B1 appeared from both screening methods (Fig. 3F). These data indicate that the CRISPRko and CRISPRa screening methods complement each other and would identify different hits in the drug transport setup.

### Drug accumulation regulated by the ABCB1 gene during transient drug exposure

**Fig. 4:**
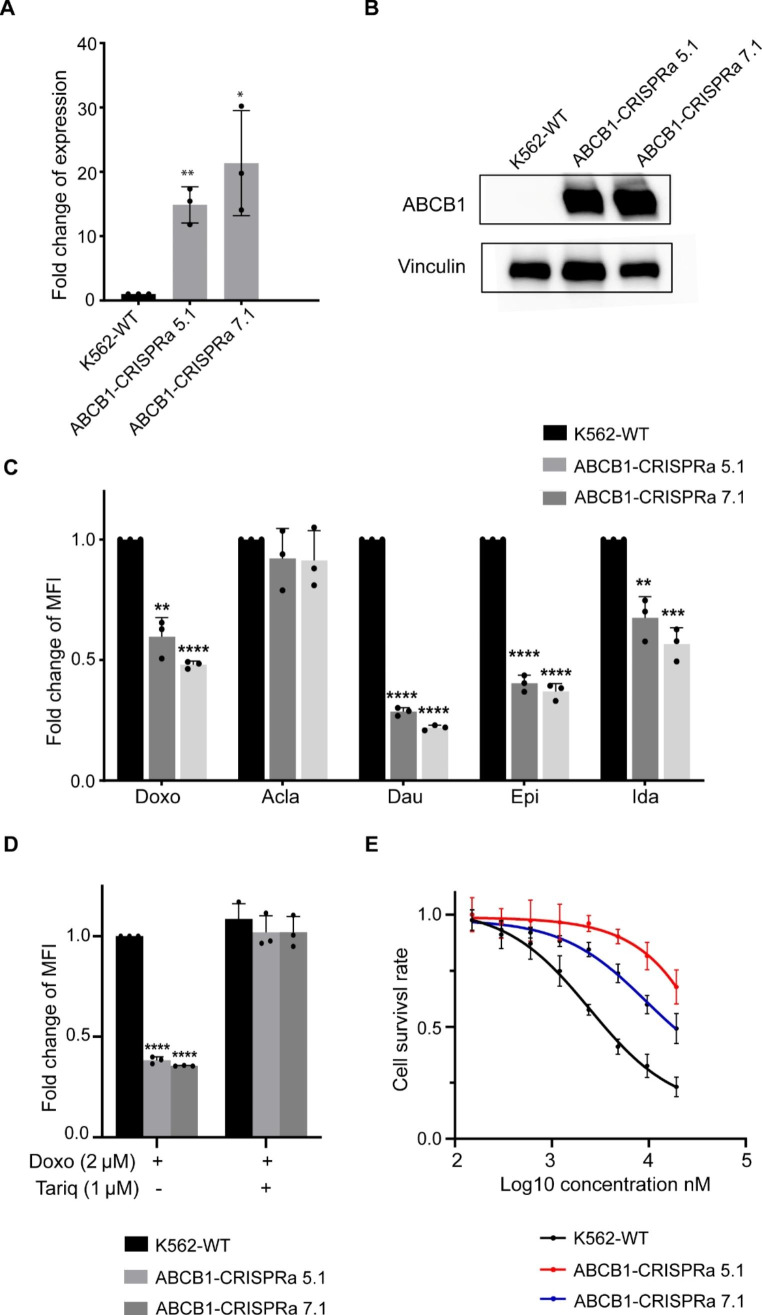
Drug accumulation regulated by the ABCB1 gene during transient drug exposure. **A.** qPCR was used to quantify the upregulation of ABCB1 gene in K562 ABCB1-CRISPRa clones. **B.** Western blotting was used to confirm the expression of ABCB1 in K562 ABCB1-CRISPRa clones. Vinculin was used as the loading control. **C.** FACS was used to quantify the uptake of drugs in K562 ABCB1-CRISPRa clones. For each group, cells were treated with doxorubicin, aclarubicin, daunorubicin, epirubicin, and idarubicin respectively at the final concentration of 2 µM for 2 h. Then fluorescence intensity of the drugs was quantified by FACS. **D.** The specific ABCB1 inhibitor tariquidar blocked the reduced uptake of doxorubicin in K562 ABCB1-CRISPRa clones. Cells were pre-treated with 1 µM tariquidar for 2 h, and then treated with 2 µM doxorubicin for 2 h. The fluorescence intensity of doxorubicin was quantified by FACS. **E.** CellTiter-Blue assay was used to quantify the cell viability. Cells were exposed to a serial dilution of doxorubicin for 72 h, then the live cells were measured. Bars show mean value ± s.e.m. (n = 2 or 3). *p < 0.05, **p < 0.005, and ***p < 0.0001 (versus the control), calculated using Student’s t-test

To show that our proposed experimental setup would reliably identify players in drug transport, the ABCB1 gene, one of the top hits from both screenings, was chosen for further analyses. Despite the fact that the ABCB1 gene has been proposed to play a role in drug exporting, the majority of the genetic perturbation screenings were done during a long period of drug exposure [34], making it interesting to see the effect of ABCB1 manipulation during transient drug exposure. As the ABCB1 gene is lowly expressed before drug exposure, we chose to use the CRISPRa system to upregulate the gene expression (Fig. 4A). Two guide RNAs were designed to target the promoter region of the ABCB1 gene, and more than 15-fold upregulation of gene expression was achieved as measured by qPCR (Fig. 4A). We also confirmed that the protein level of the ABCB1 gene was elevated by the two respective CRISPRa guide RNAs (Fig. 4B).

As the result of the upregulation of the ABCB1 gene, the direct uptake of doxorubicin monitored by FACS was reduced by 50% in the two respective clones (Fig. 4C). It is interesting to see that not only the direct uptake of doxorubicin, but that of other clinically used anthracycline drugs such as daunorubicin, epirubicin and idarubicin was also reduced, except for aclarubicin (Fig. 4C and Supplementary Fig. 3). These data indicate that aclarubicin would serve as an alternative anthracycline to overcome the drug resistance caused by the upregulation of the ABCB1 gene. To further confirm that the reduced uptake of doxorubicin measured by FACS was directly caused by CRISPRa-mediated upregulation of ABCB1, a specific ABCB1 inhibitor tariquidar was used to treat the CRISPRa clones. No reduction of doxorubicin uptake was observed in tariquidar-treated clones expressing CRISPRa activating the ABCB1 gene (Fig. 4D), suggesting that the change of doxorubicin transport was not induced by the off-target effects of CRISPRa system. As a result, K562 clones with CRISPRa-mediated upregulation of ABCB1 gene became more resistant to doxorubicin and other anthracycline drugs compared to the parental K562 cells (Fig. 4E and Supplementary Fig. 4).

### SLC2A3 gene serving as a novel doxorubicin importer and response marker

Only limited research has been done to identify drug importers of doxorubicin over the past several decades, and the contribution of these factors to drug response is still obscure [37]. Recently, a CRISPRko screening effort has been attempted to identify potential importers for many drugs, including doxorubicin. However the screening readout was based on cell survival but not direct drug uptake, and the CRISPRko screening system lacks the resolution to identify lowly expressed genes as potential functional hits[8]. Therefore, CRISPRa screenings that upregulate potential genes may identify additional and novel hits that were overlooked before, as we also observed (Fig. 3F). SLC2A3 gene was the top hit as a drug importer of doxorubicin from our CRISPRa screenings, which studied doxorubicin uptake directly (Fig. 3C and D). SLC2A3 encodes the solute carrier transmembrane glucose transporter 3 (GLUT3) that binds glucose and facilitates glucose uptake [38]. GLUT3 is expressed in neuronal tissues, as well as heart and white blood tissues [39]. Some tumors such as glioblastoma and triple-negative breast cancer may rely on the overexpression of GLUT3 and hence have an addition to it, making it a vulnerable drug target [40, 41].

**Fig. 5:**
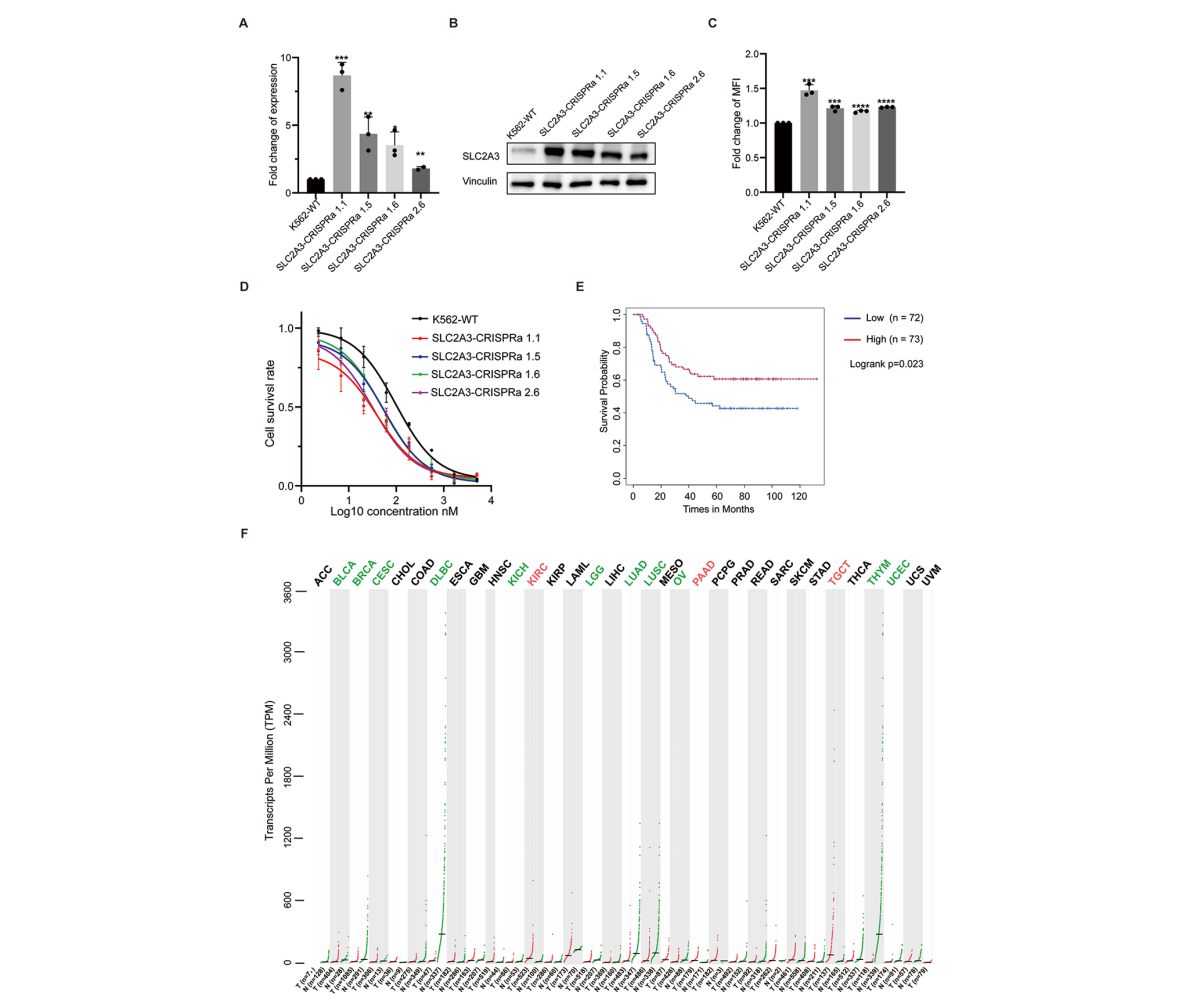
SLC2A3 gene serving as a novel doxorubicin importer and response marker. **A.** qPCR was used to quantify the upregulation of SLC2A3 gene in K562 SLC2A3-CRISPRa clones. **B.** Western blotting was used to confirm the expression of SLC2A3 in SLC2A3-CRISPRa clones. Vinculin was used as the loading control. **C.** Quantification of doxorubicin uptake in K562 SLC2A3-CRISPRa clones. Cells were treated with doxorubicin at the final concentration of 2 µM for 2 h. Then the fluorescence intensity of the drugs was quantified by FACS. **D.** CellTiter-Blue assay was used to measure the cell viability. Cells were exposed to serial dilutions of doxorubicin for 72 h, then the live cells were measured. Bars show mean value ± s.e.m. (n = 2 or 3). *p < 0.05, **p < 0.005, and ***p < 0.0001 (versus the control), calculated using Student’s t-test. **E.** Kaplan-Meier analysis of overall survival of AML patients from TARGET study based on the SCL2A3 expression level. The red line indicates survival probability from the AML patients with higher expression of SLC2A3 (n = 73). The blue line indicates survival probability from the AML patients with lower expression of SLC2A3 (n = 72). The p value was calculated using the log rank test. **F.** Dot plot showing SLC2A3 expression across 33 different cancer types and their paired normal tissues. The red dots represent normalized SLC2A3 expression of samples from specific cancer type. The green dots represent normalized SLC2A3 expression of samples from specific normal tissue type. The cancer type with higher SLC2A3 expression than its matched normal tissue is indicated by red. The cancer type with lower SLC2A3 expression than its matched normal tissue is indicated by green. TPM, transcripts per million; ACC, adrenocortical carcinoma; BLCA, Bladder urothelial carcinoma; BRCA, breast invasive carcinoma; CESC, cervical squamous cell carcinoma and endocervical adenocarcinoma; CHOL, cholangio carcinoma; COAD, colon adenocarcinoma; DLBC, lymphoid neoplasm diffuse large B-cell lymphoma; ESCA, esophageal carcinoma; GBM, glioblastoma multiforme; HNSC, head and neck squamous cell carcinoma; KICH, kidney chromophobe; KIRC, kidney renal clear cell carcinoma; KIRP, kidney renal papillary cell carcinoma; LAML, acute myeloid leukemia; LGG, brain lower grade glioma; LIHC, liver hepatocelular carcinoma; LUAD, lung adenocarcinoma; LUSC, lung squamous cell carcinoma; MESO, mesothelioma; OV, ovarian serous cystadenocarcinoma; PAAD, pancreatic adenocarcinoma; PCPG, pheochromocytoma and paraganglioma; PRAD, prostate adenocarcinoma; READ, rectum adenocarcinoma; SARC, sarcoma; SKCM, skin cutaneous melanoma; STAD, stomach adenocarcinoma; TGCT, testicular germ cell tumors; THCA, thyroid carcinoma; THYM, thymoma; UCEC, uterine corpus endometrial carcinoma; UCS, uterine carcinosarcoma; UVM, uveal melanoma

To confirm the role of SLC2A3 in mediating doxorubicin uptake, we generated 4 independent clones using 2 different guide RNAs targeting the promoter region of SLC2A3 using CRISPRa system. A robust upregulation of SLC2A3 was achieved from all these clones, as measured by qPCR (Fig. 5A). As a result, the protein levels of SLC2A3 were also increased (Fig. 5B). It is noteworthy that the protein level of SLC2A3 was very low in the parental K562 cells, pointing out that screening strategies to knockdown or knockout this gene would not be possible to identify this gene as a potential doxorubicin importer.

We then monitored the direct uptake of doxorubicin of these clones, as measured by FACS. Up to 50% increase in doxorubicin accumulation was observed in these clones compared to the parental K562 cells when cells were transiently exposed to doxorubicin (Fig. 5C and Supplementary Fig. 5). SLC2A3-mediated doxorubicin uptake was also observed in 293T cells, indicating a general role of this gene in the uptake of doxorubicin (Supplementary Fig. 6). We further determined the effects of the increase of drug accumulation, and observed a significantly elevated cell killing in the SLC2A3 upregulating clones compared to the parental K562 cells when exposed to doxorubicin (Fig. 5D), suggesting a potentially important role of SLC2A3 in tumors responding to doxorubicin treatment. Indeed, in some of the AML patients, the expression level of SLC2A3 is higher, and when the contribution of SLC2A3 was considered in the overall survival of AML patients from the TARGET study, potential better survival effects were seen in patients with higher SLC2A3 expression (Fig. 5E) [42]. Because only a subgroup of these AML patients might have received doxorubicin treatment, it is possible that the contribution of SLC2A3 benefiting patients’ response to the doxorubicin-containing regimen is even stronger. At the same time, these data also suggest that the SLC2A3 gene could be a predictive marker of tumors responding to doxorubicin treatment. The expression level of SLC2A3 is higher in tumors such as kidney renal clear cell carcinoma (KIRC), pancreatic adenocarcinoma (PAAD), and testicular germ cell tumors (TGCT), compared to the matching healthy tissues (Fig. 5F) [43], indicating these patients with higher SLC2A3 expression might also benefit from doxorubicin containing regimen. Indeed, previous studies showed that patients with testicular germ cell tumors may benefit from doxorubicin treatment in clinical trials [44], and future clinical trials may be warranted to test doxorubicin in treating subgroups of patients with tumors expressing elevated SLC2A3.

## Discussion

The uptake and export of drugs by different cells and tissues represent one of the first factors affecting drug responses. The contribution of drug exporters, exemplified by ABCB1 in multi-drug resistance, has been characterized for a long time. In preclinical studies for new drugs, testing the effect of these exporters on drugs is also one of the requirements for subsequent filing of clinical trials, highlighting the importance of drug transporters in drug development. However, so far, systematic studies on the uptake of different drugs have been very limited, and often cell survival rather than direct uptake was used as the readout [8]. Furthermore, only CRISPR-mediated knockout was used to identify potential transporters, limiting the resolution to identify potential drug importers with lower expression in a defined cell type. We designed custom CRISPRko (for gene knockout) and CRISPRa (for gene activation) libraries targeting all potential membrane-associated transporters and proteins; furthermore, we monitored the direct uptake of drugs by FACS as the screening readout for the identification of transporters/proteins directly involved in drug uptake. Using this rationale, we were able to identify both known drug export transporter genes such as ABCB1 and ABCG2, and novel doxorubicin import transporter gene SLC2A3 for a broadly used chemotherapeutic drug doxorubicin. In addition, we found that CRISPRa screening would identify novel drug transporters, which would be missed by CRISPRko methods. This is especially important, as often genetic screenings are performed in one or limited cell or tissue types, in which the expression level of many genes is low (Supplementary Fig. 1). The upregulation of the novel doxorubicin importer SLC2A3 would directly increase the uptake of doxorubicin in K562 and 293T cells (Fig. 5C and Supplementary Fig. 6), and result in the better killing of these tumor cells. These data indicate that SLC2A3 could be a potential marker for identifying patients who may benefit from doxorubicin treatment. Furthermore, as GLUT3, the protein product of SLC2A3, is also present in heart tissues [45], and heart damage is still one of the major side effects associated with doxorubicin [19], SLC2A3 (GLUT3) may also contribute to doxorubicin-mediated cardiotoxicity. GLUT3 could be a novel target to reduce cardiotoxicity.

## Electronic supplementary material

Below is the link to the electronic supplementary material.

Supplementary Material 1
