# Supplementary material for: Targeted CRISPR activation and knockout screenings identify novel doxorubicin transporters
Source: Cell Oncol (Dordr). 2023 Jul 31;46(6):1807–20. doi: 10.1007/s13402-023-00847-0 (PMC10698112; doi:10.1007/s13402-023-00847-0)
Supplement: Supplementary file 1 — Supplementary Material 1 [file 13402_2023_847_MOESM1_ESM.docx]

**
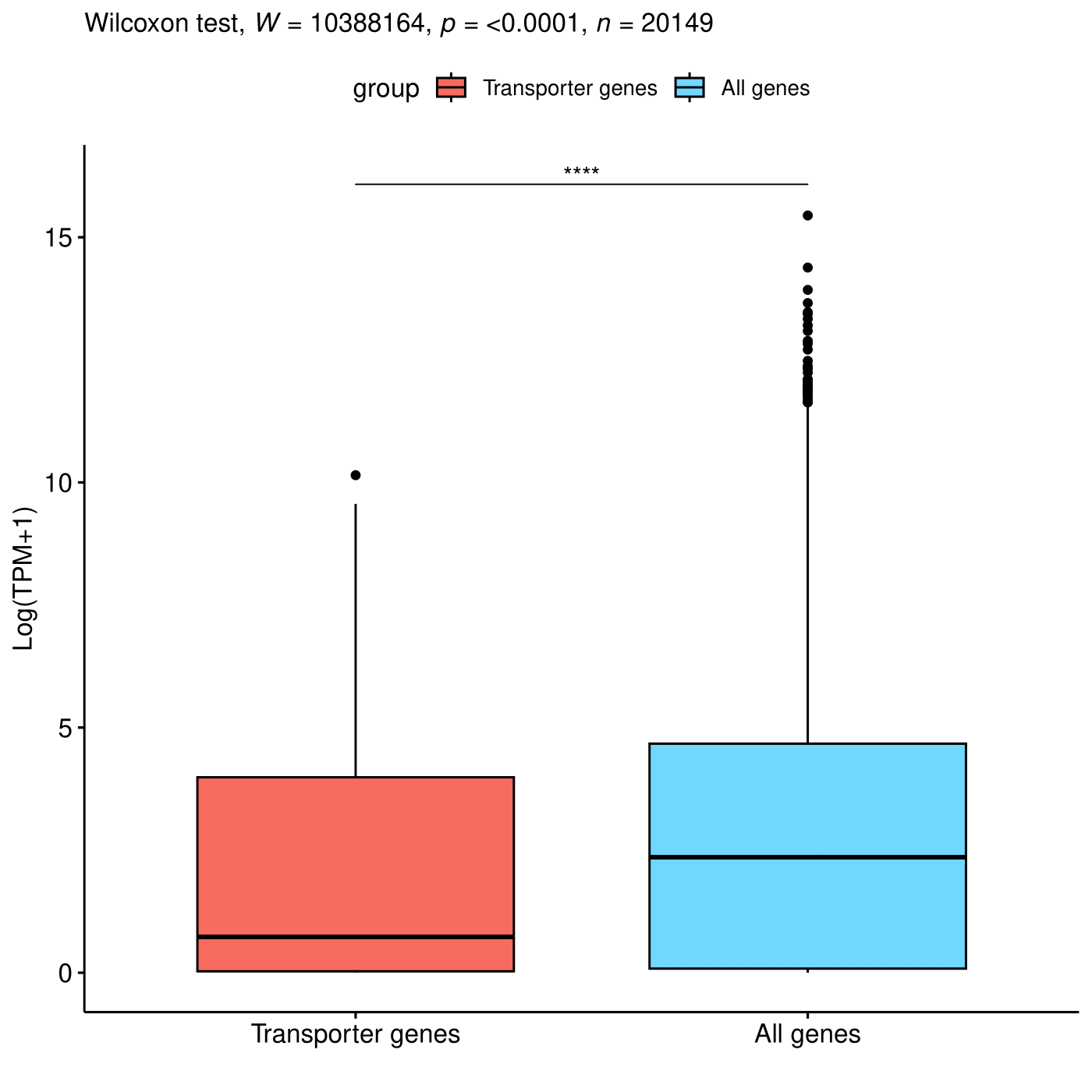
**

**Supplementary Fig. 1**: Gene expression of transporter genes compared to all genes. The *y* axis represents the logarithm of TPM normalized expression level of genes plus 1. The p value was calculated using unpaired two-sided Wilcoxon Rank Sum test. n = 979 (only transporter) or 19,193 (all) genes. K562 RNA-seq data were downloaded from Dependency Map Portal. ****p < 0.0001.


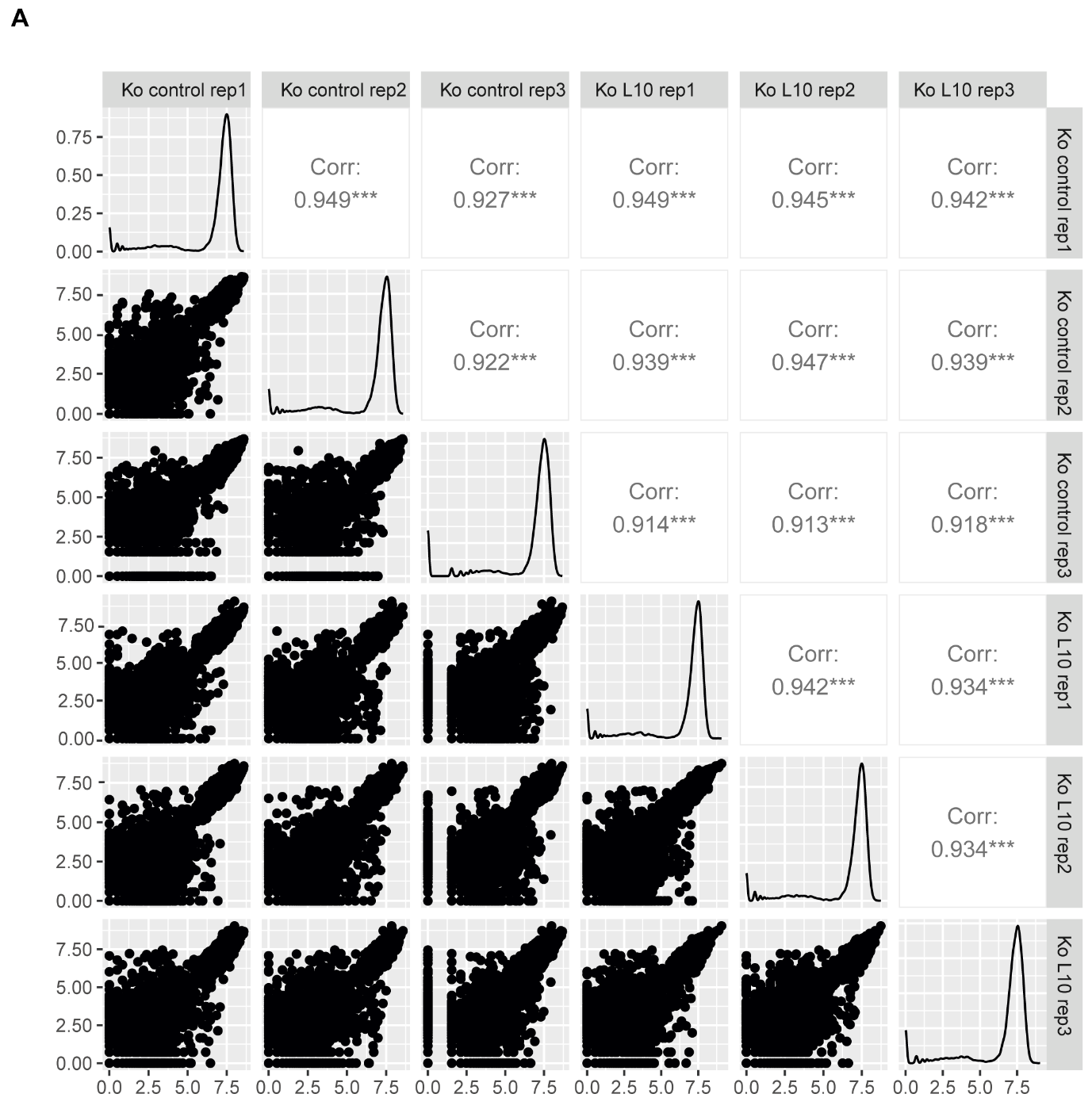


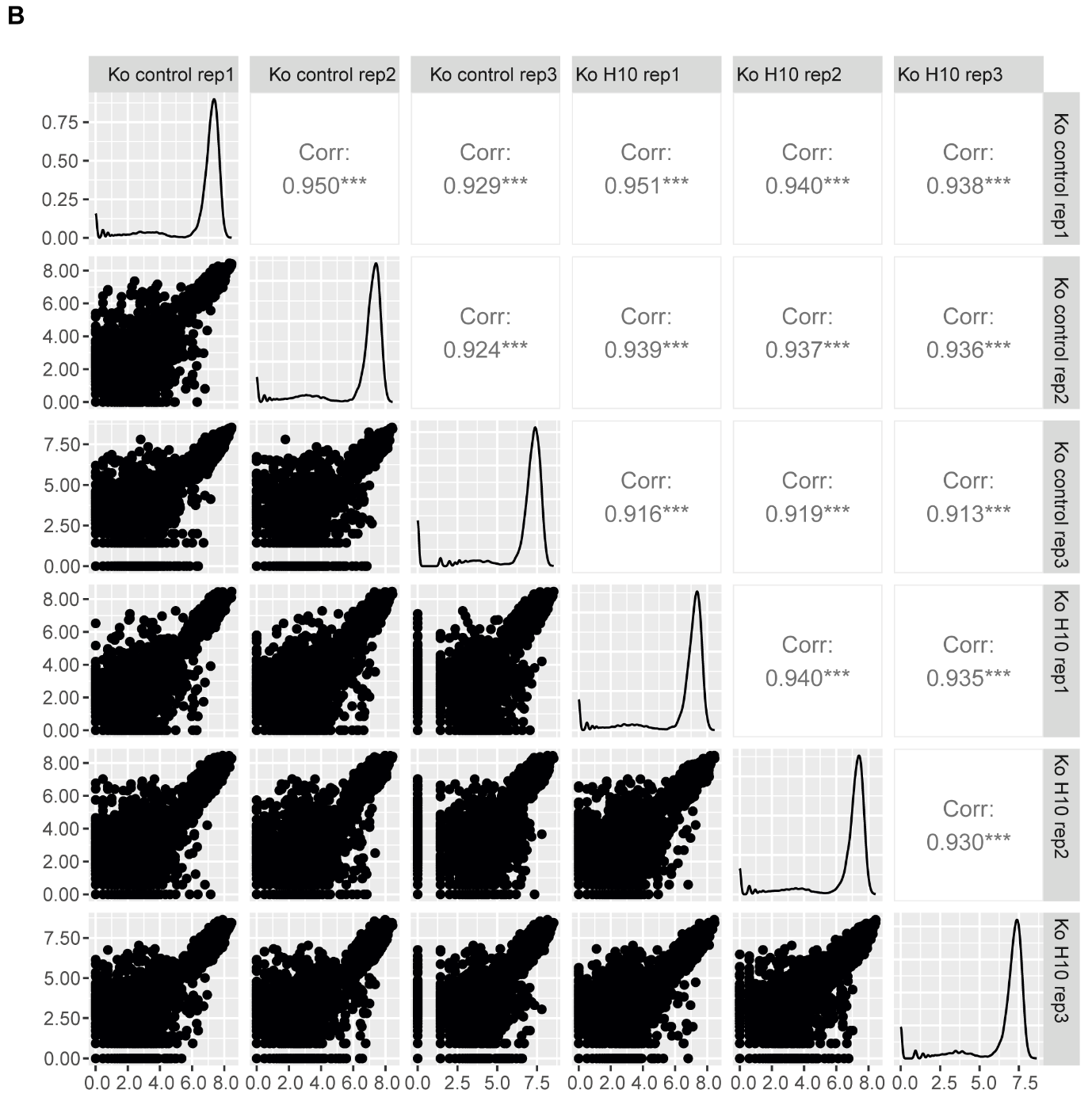


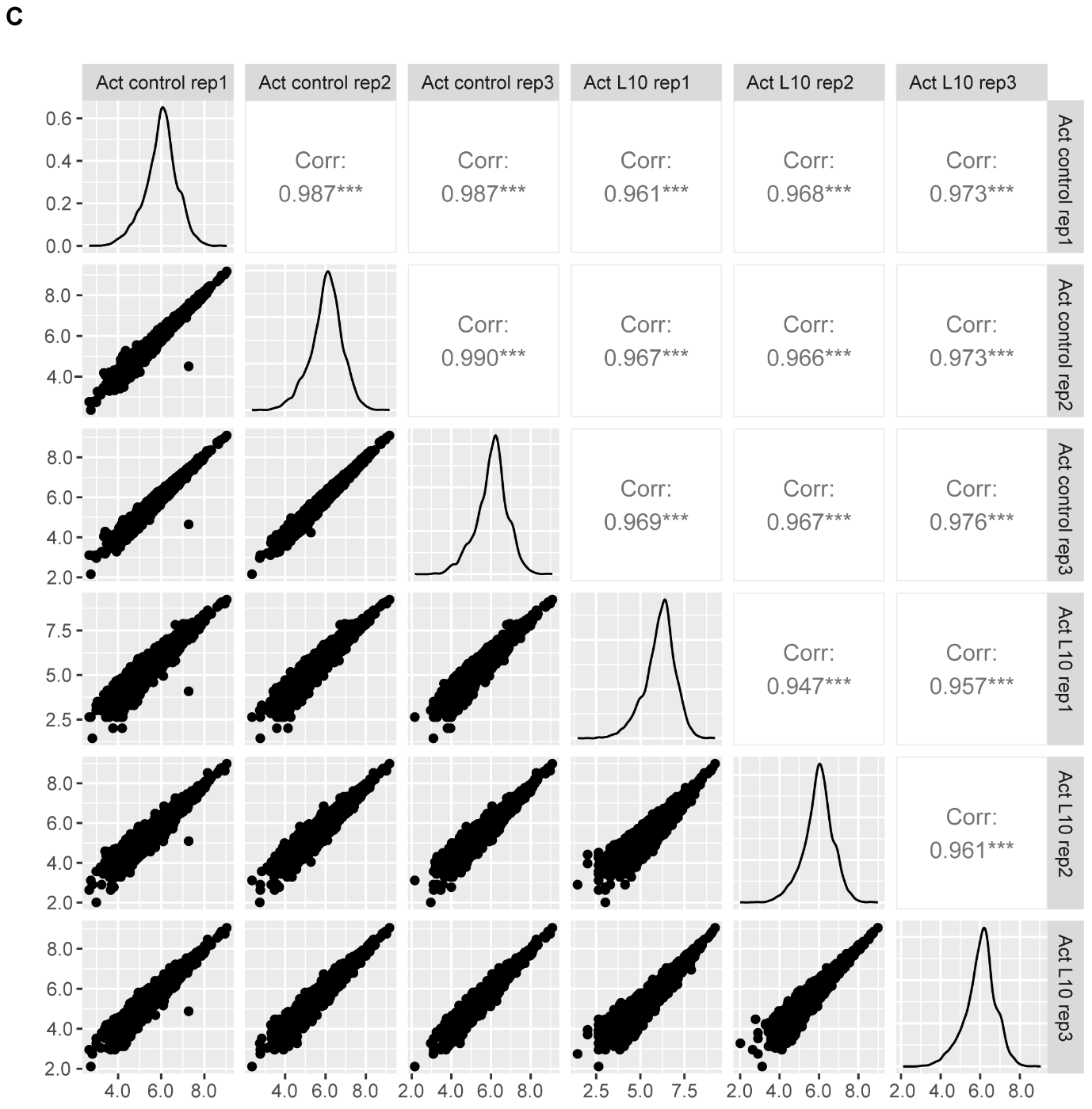


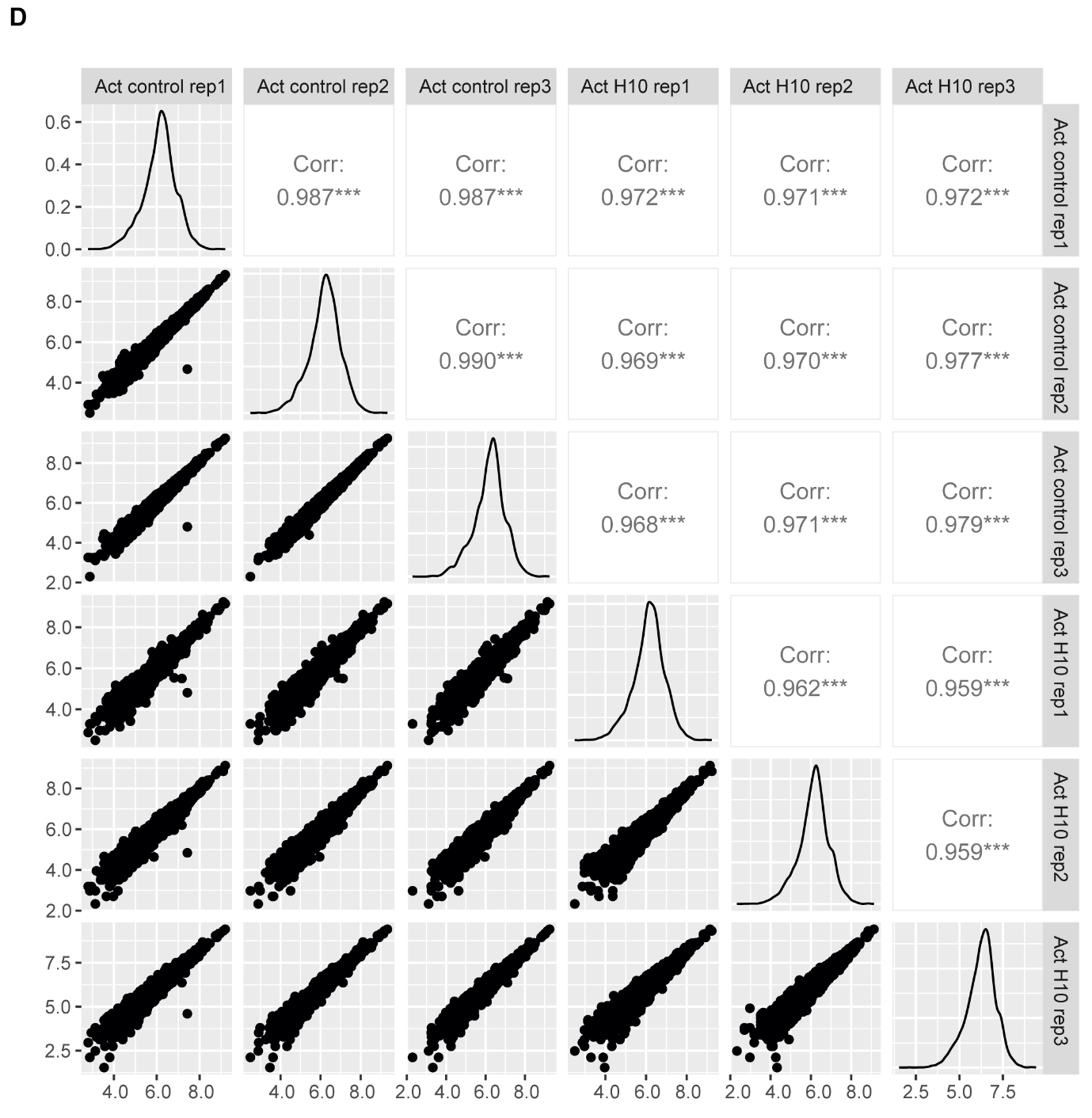


**Supplementary Fig. 2**: Pairwise correlation of CRISPRko and CRISPRa screen replicates. **A**. Pairwise correlation of L10 population from the CRISPRko screenings with the control group. **B**. Pairwise correlation of H10 population from the CRISPRko screenings with the control group. **C**. Pairwise correlation of L10 population from the CRISPRa screenings with the control group. **D**. Pairwise correlation of H10 population from the CRISPRa screenings with the control group. The logarithm of read count plus 1 of each replicate was plotted. Pearson correlation coefficient was calculated to measure the correlation between each replicate pair.


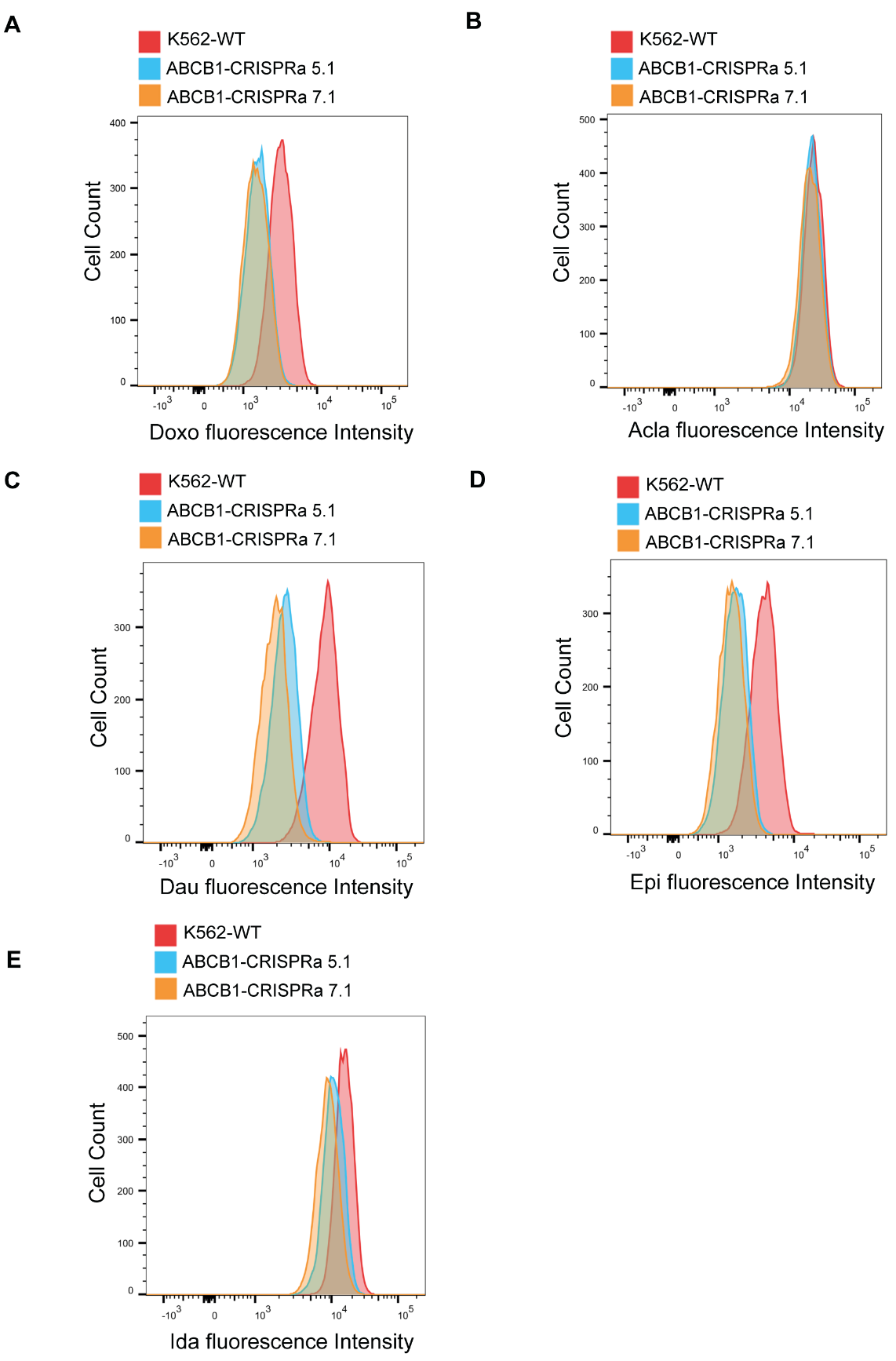


**Supplementary Fig. 3**: Drug accumulation regulated by the ABCB1 gene during transient drug exposure. **A-E**. FACS histograms from the quantification of the drug uptake in K562 ABCB1-CRISPRa clones. For each group, cells were treated with doxorubicin (A), aclarubicin (B), daunorubicin (C), epirubicin (D), and idarubicin (E) respectively at the final concentration of 2 μM for 2 h. Then fluorescence intensity of the drugs was quantified by FACS.


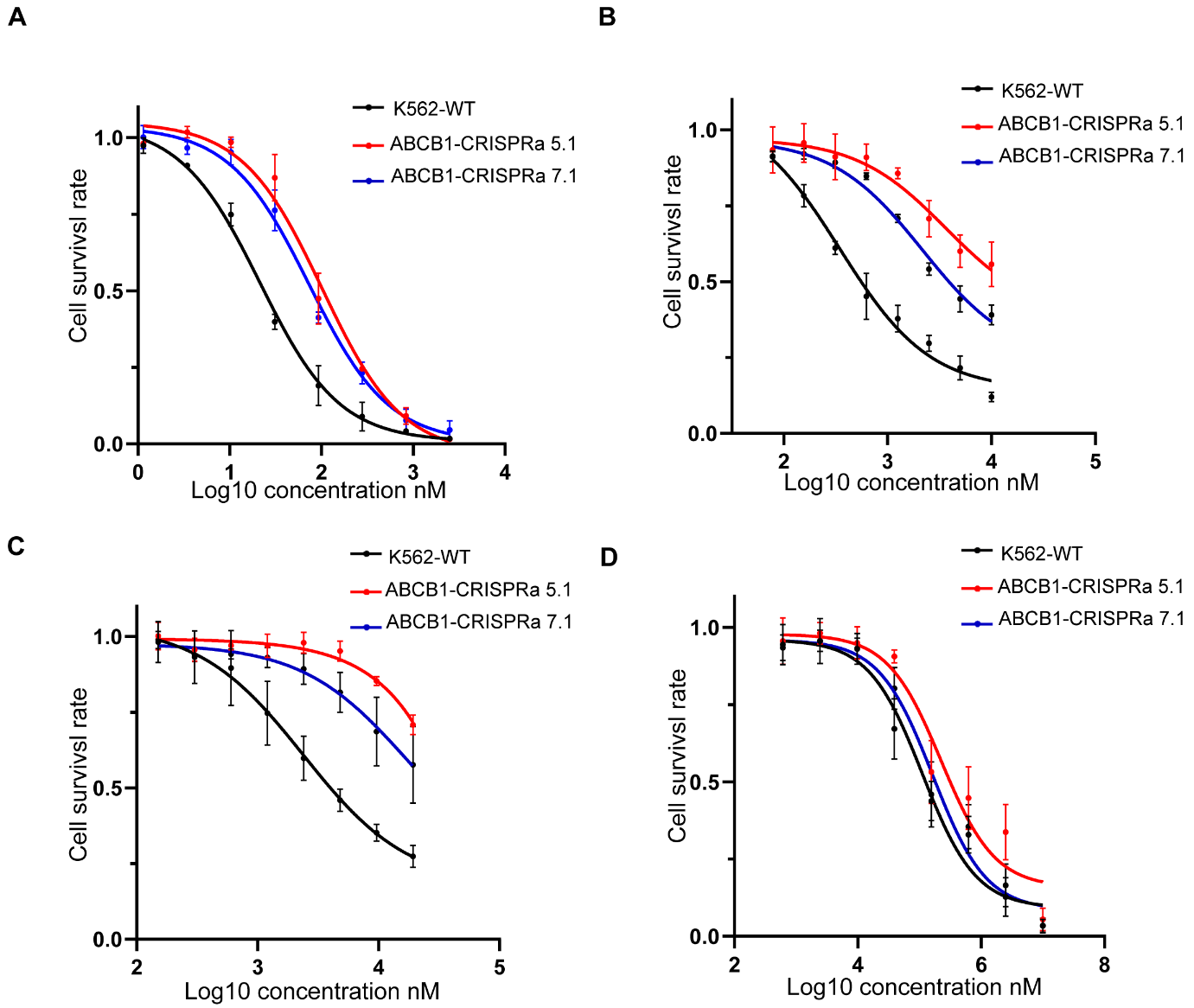


**Supplementary Fig. 4**: Cell survival regulated by the ABCB1 gene. For each group, cells were treated with aclarubicin, daunorubicin, epirubicin, and idarubicin respectively at a serial dilution of drugs for 72 h. CellTiter-Blue assay was used to quantify the cell viability. **A**. Cells were exposed to a serial dilution of aclarubicin for 72 h, then the live cells were measured. **B**. Cells were exposed to a serial dilution of daunorubicin for 72 h, then the live cells were measured. **C**. Cells were exposed to a serial dilution of epirubicin for 72 h, then the live cells were measured. **D**. Cells were exposed to a serial dilution of idarubicin for 72 h, then the live cells were measured.


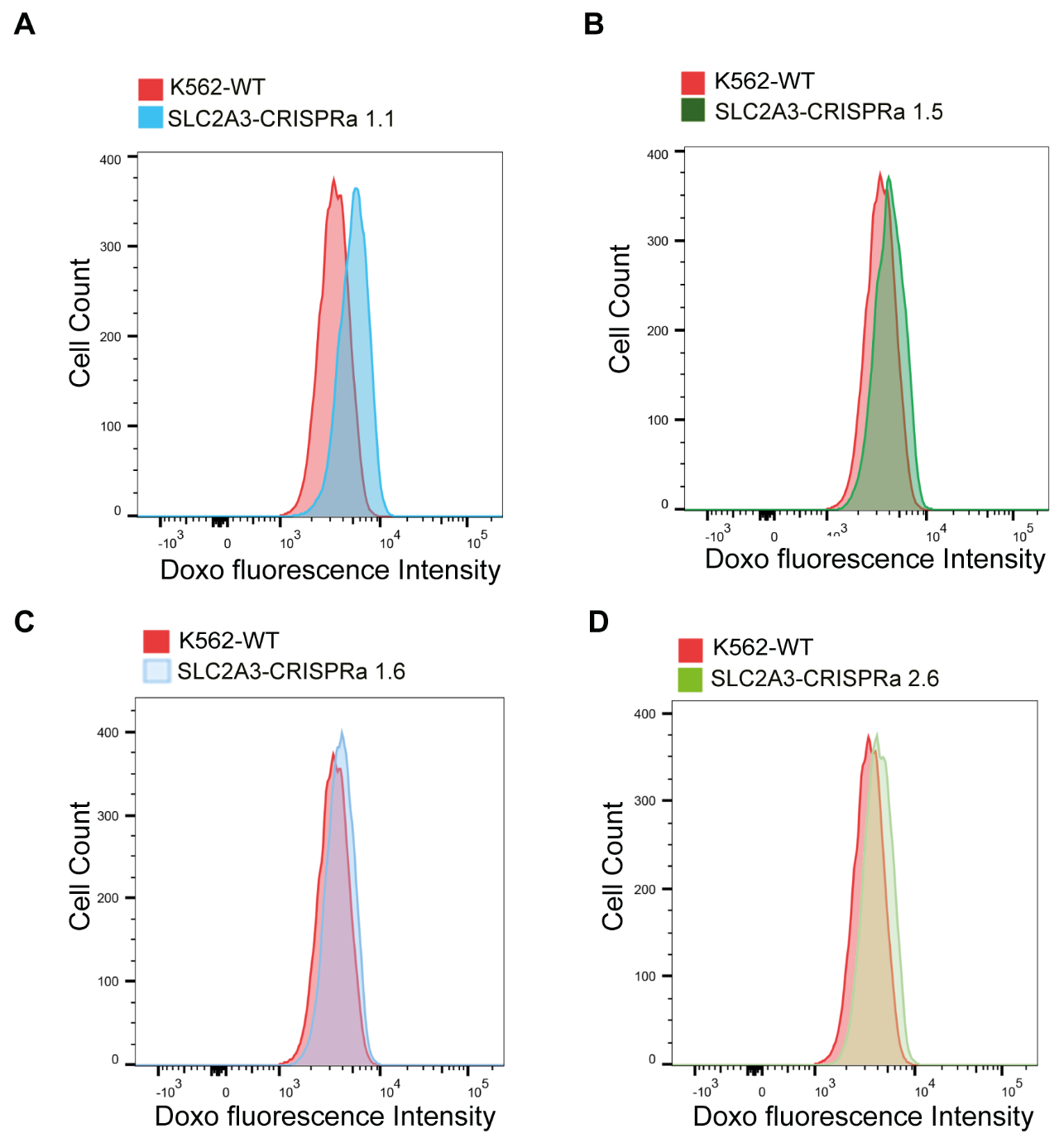
**Supplementary Fig. 5**: Drug accumulation regulated by the SLC2A3 gene during transient doxorubicin exposure. **A-D**. FACS histograms from the quantification of the drug uptake in K562 SLC2A3-CRISPRa clones. For each clone, cells were treated with doxorubicin at the final concentration of 2 μM for 2 h. Then fluorescence intensity of the doxorubicin was quantified by FACS.


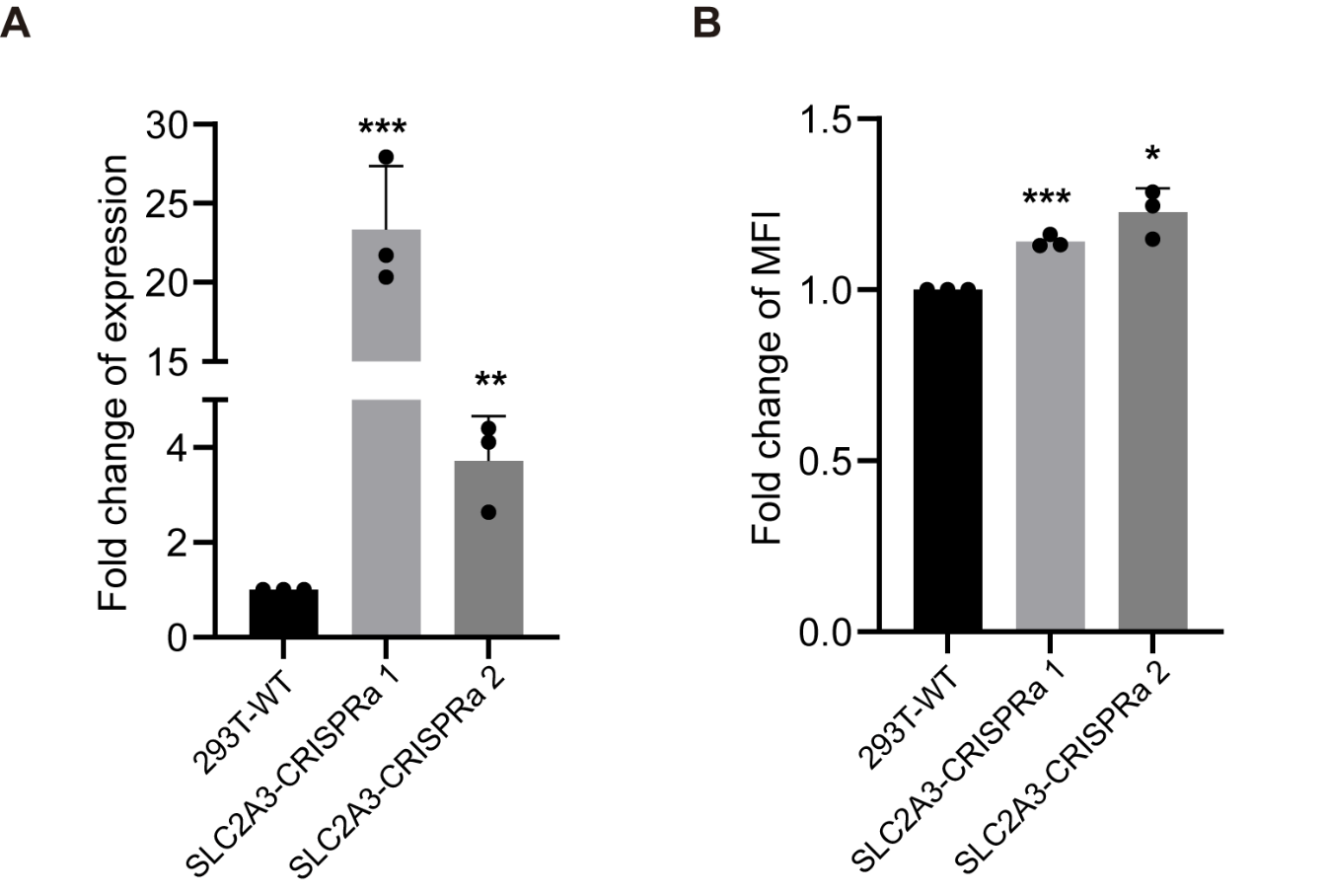


**Supplementary Fig. 6**: Doxorubicin uptake was enhanced in 293T cells with elevated SLC2A3 expression. **A**. qPCR was used to quantify the expression of SLC2A3 in 293T SLC2A3-CRISPRa bulk cells. **B.** FACS was used to quantify the uptake of doxorubicin in 293T SLC2A3-CRISPRa bulk cells. For each cell line, cells were treated with doxorubicin at the final concentration of 2 μM for 2 h. The fluorescence intensity of the drugs was quantified by FACS. Bars show mean value ± s.e.m. and significance was calculated using Student’s t-test (n = 2 or 3). *p < 0.05, **p < 0.005, and ***p < 0.0001 (versus the 293T-WT cells).
